# Ultrasound-Assisted Formulation of a High-DHA Omega-3 Concentrate: Physical and Oxidative Stability of Oil-in-Water Emulsions

**DOI:** 10.3390/foods15162807

**Published:** 2026-08-11

**Authors:** Esther de Paz, Óscar Benito-Román, María Teresa Sanz, Rodrigo Melgosa, Ángela G. Solaesa, Sagrario Beltrán, José Manuel Benito

**Affiliations:** 1 Department of New Food Ingredients, CNTA (National Center of Food Technology and Safety), Crta-Na134, km 53, 31570 San Adrián, Spain; edepaz@cnta.es; 2 Department of Biotechnology and Food Science, Chemical Engineering Section, Faculty of Sciences, University of Burgos, Plaza Misael Bañuelos s/n, 09001 Burgos, Spain; tersanz@ubu.es (M.T.S.); rmgomez@ubu.es (R.M.); beltran@ubu.es (S.B.); 3 Department of Anatomy and Radiology, Faculty of Health Sciences, University of Valladolid, Campus of Soria, 42004 Soria, Spain; angela.garcia.solaesa@uva.es

**Keywords:** oil-in-water emulsions, omega-3 polyunsaturated fatty acids, oxidation stability, antioxidants

## Abstract

Omega-3 (n-3) polyunsaturated fatty acids (PUFA) are associated with several health benefits, but their high susceptibility to lipid oxidation and low water dispersibility limit their incorporation into food products. In this study, an oil-in-water emulsion was developed from a commercial fish-oil concentrate containing 92% *w*/*w* total n-3 PUFA and 73% *w*/*w* docosahexaenoic acid (DHA), using octenyl succinic anhydride (OSA)-modified starch as the sole emulsifying carrier. Response surface methodology was used to evaluate the effects of oil content, ultrasound emulsification time and ultrasound amplitude on the volume-weighted mean droplet diameter, D[4,3]. The selected formulation, prepared with an oil content of 20% *w*/*w* relative to the total solids, an emulsification time of 180 s, and an ultrasound amplitude of 100%, exhibited a D[4,3] of 121 ± 1 nm. During storage at ambient temperature in darkness, the emulsion maintained low peroxide values, ranging from 1.2 to 1.4 meq O_2_/kg oil, and thiobarbituric acid reactive substances (TBARS) values between 1.3 and 2.1 mmol/kg oil during the first 5 days, indicating that the formulation delayed lipid oxidation of the n-3 PUFA concentrate. The addition of α-tocopherol did not improve oxidative stability compared with the control emulsion. In contrast, ascorbic acid maintained low peroxide values and reduced TBARS accumulation during storage, with values lower than those of the control emulsion and close to the initial TBARS value of the n-3 PUFA concentrate. These results indicate that, under the conditions evaluated, ascorbic acid was more effective than α-tocopherol in delaying the formation of secondary lipid oxidation products.

## 1. Introduction

The demand for long-chain omega-3 polyunsaturated fatty acids (n-3 PUFA), particularly eicosapentaenoic acid (EPA) and docosahexaenoic acid (DHA), has significantly increased due to their recognized health benefits, especially in cardiovascular protection and the prevention of inflammatory disorders [1]. Although EPA and DHA can be endogenously synthesized from α-linolenic acid (ALA) found in plant sources such as linseed and chia, the conversion rate is very limited (5–10% for EPA and 1–5% for DHA). For this reason, EPA and DHA are considered essential fatty acids that must be obtained directly from the diet [2]. Since modern dietary patterns are typically characterized by high saturated fat intake and low polyunsaturated fatty acid consumption, nutritionists recommend increasing the intake of fish and green vegetables. However, dietary habits are difficult to modify; consequently, a wide range of products enriched with n-3 PUFA, including nutritional supplements and functional foods, has been developed to help bridge this nutritional gap [3]. Despite these advances, the incorporation of omega-3 fatty acids into food matrices remains challenging because of their high susceptibility to lipid oxidation. Exposure to oxygen, elevated temperatures, and moisture leads to the formation of primary (hydroperoxides) and secondary (aldehydes, ketones, volatile compounds) oxidation products, which cause rancidity, nutrient degradation, and undesirable sensory changes, ultimately reducing consumer acceptance and product shelf life [4,5,6].

To mitigate these challenges, different stabilization strategies have been explored. Conventional approaches include the addition of natural or synthetic antioxidants and improved packaging systems. More advanced strategies are based on encapsulation technologies, particularly emulsions and nanoemulsions [7], which provide interfacial protection and limit the exposure of omega-3 fatty acids to pro-oxidant environments [8]. Emulsion-based delivery systems have attracted considerable attention as they not only enhance oxidative stability but also improve bioavailability and sensory characteristics, with nanoemulsions emerging as especially promising to overcome the stability and bioavailability limitations of omega-3 fatty acids in functional foods [9,10,11].

Among available emulsification technologies, ultrasound has gained significant attention. Ultrasound processing is recognized for its energy efficiency and ability to produce emulsions with superior encapsulation efficiency and fine droplet size distributions compared to conventional methods [12,13]. The cavitation phenomenon generated during sonication creates intense shear forces, turbulence and microjets that reduce oil-droplet size to the nanoscale, leading to emulsions with narrow size distributions and improved kinetic stability [14]. Ultrasound emulsification operates at relatively low bulk temperatures, which is beneficial for heat-sensitive PUFAs, and can be applied in both batch and continuous modes. By generating uniform droplets and improving oil dispersion, it enhances both physical stability and bioavailability [9]. However, cavitation can also induce the formation of reactive species, potentially influencing oxidative pathways; therefore, careful optimization of process parameters such as power, time, duty cycle, and temperature is essential [14].

Recent studies have confirmed the feasibility of preparing stable omega-3 emulsions and nanoemulsions using ultrasound. Lane et al. [15] demonstrated that algal oil nanoemulsions stabilized with natural emulsifiers retained their fatty acid composition during storage under moderate conditions, though instability increased at accelerated temperatures. Similarly, Zhou et al. [16] reported that ultrasound-assisted nanoemulsions prepared with lecithin and Tween 40 exhibited both enhanced oxidative stability and higher digestibility compared to bulk oil. Other studies have evaluated the incorporation of antioxidants into fish oil emulsions to further delay oxidation. For example, Serfert et al. [17] showed that adding a combination of tocopherols, ascorbyl palmitate, lecithin, and rosemary extract delayed oxidation in fish oil emulsions, while Baik et al. [18] reported improved stability of microencapsulated fish oil when α-tocopherol was added at concentrations above 250 ppm.

Starch sodium octenyl succinate (OSA-modified starch) was selected as the emulsifying carrier because of its amphiphilic character and its demonstrated suitability for the encapsulation and delivery of flavors, carotenoids, vitamins, and oils rich in polyunsaturated fatty acids [17,19,20,21,22,23,24,25]. OSA-modified starch is authorized as a food additive in the European Union under the designation E1450, subject to the conditions of use established for the corresponding food categories [26]. The European Food Safety Authority concluded that there was no safety concern for modified starches, including E1450, at the reported uses and use levels [27].

Although OSA-modified starch and ultrasound-assisted emulsification have previously been applied to different lipid delivery systems [28,29,30], the specific contribution of the present study lies in their combined application to a commercial fish-oil concentrate containing 92% *w*/*w* total n-3 PUFA and 73% *w*/*w* DHA. The emulsion was formulated using OSA-modified starch as the sole emulsifying carrier, without additional low-molecular-weight surfactants, and was systematically optimized by response surface methodology as a function of oil content, ultrasound emulsification time, and ultrasound amplitude. The physical stability of the selected formulation was subsequently evaluated by laser diffraction and static multiple light scattering, and the effects of a hydrophilic antioxidant, ascorbic acid, and a lipophilic antioxidant, α-tocopherol, on primary and secondary lipid oxidation, were compared. Therefore, the aim of this study was to develop and characterize an ultrasound-assisted oil-in-water emulsion containing a high-DHA omega-3 concentrate and to evaluate its physical and oxidative stability during storage.

## 2. Materials and Methods

### 2.1. Materials

A commercial omega-3 polyunsaturated fatty acid (n-3 PUFA) concentrate (Algatrium™ Plus) was provided by Brudy Technology S.L. (Barcelona, Spain). According to information supplied by the manufacturer, the concentrate was derived from fish oil and produced using a proprietary enzymatic process designed to obtain triacylglycerols enriched in DHA, with a high proportion of DHA at the sn-2 position of the glycerol backbone. The fish species and the specific procedure used for the initial extraction of the crude fish oil were not disclosed by the supplier and were not part of the experimental work conducted in this study. The lipid-class distribution and fatty-acid composition of the commercial concentrate were experimentally determined as described in Section 2.2.

OSA-modified starch refined from waxy maize was kindly supplied by Ingredion (Hamburg, Germany). Hydrochloric acid (37%), trichloroacetic acid (99.8%), 2-propanol, methanol and butanol were purchased from VWR Chemicals (Darmstadt, Germany). Iron (II) sulfate heptahydrate, 2-thiobarbituric acid, and ammonium thiocyanate were supplied by Merck KGaA (Darmstadt, Germany). α-Tocopherol, cumene hydroperoxide (80%), and 1,1,3,3-tetraethoxypropane (≥96%) were purchased from Sigma-Aldrich (St. Louis, MO, USA). Barium chloride dihydrate and 2,2,4-trimethylpentane were supplied by Panreac Química SLU (Castellar del Vallès, Spain) and Macron Fine Chemicals™ (Radnor, PA, USA), respectively. Milli-Q water (Millipore, St. Louis, MO, USA) was used for the preparation of all samples.

### 2.2. n-3 PUFA Concentrate Characterization

The distribution of neutral lipid classes in the n-3 PUFA concentrate was established by normal-phase HPLC. Fatty acid ethyl esters, monoacylglycerides, diacylglycerides, and triacylglycerides were quantified using the chromatographic and calibration procedure previously reported [31].

Fatty-acid composition was determined by gas chromatography in accordance with AOAC method [32]. The instrumental conditions and analytical procedure were those reported by Rebolleda et al. [33].

The initial oxidation status of the n-3 PUFA concentrate was characterized through peroxide value (PV), *p*-anisidine value (AV), and thiobarbituric acid reactive substances (TBARS). PV and AV were determined according to standard methods [34,35]. TBARS values were quantified using the procedure described in Section 2.6.2., adapted from Elias et al. [36] and Chityala et al. [37].

### 2.3. Emulsion Preparation

A high-intensity ultrasonic homogenizer (Sonics VCX 500, 500 W, 20 kHz, Newtown, CT, USA) equipped with a 13 mm titanium-alloy probe was used to prepare the emulsions. An aqueous OSA-modified starch solution was first prepared so that the final solids content was 30% *w*/*w* in every formulation. The n-3 PUFA concentrate was added dropwise to the aqueous OSA-modified starch solution under continuous magnetic stirring. The oil content was varied between 10 and 30% *w*/*w* of the total solids, corresponding to 3–9% *w*/*w* of the final emulsion. No rotor–stator or other high-shear device was used during this pre-emulsification step. After oil addition, the mixture was magnetically stirred for 5 min at room temperature to obtain a coarse pre-emulsion. This pre-mixing step was used to disperse the two immiscible phases before sonication and thereby reduce the energy required during ultrasound emulsification [38]. The pre-emulsion was subsequently processed using ultrasound pulses of 5 s followed by 5 s pauses to limit sample heating. The effects of ultrasound emulsification time, ranging from 60 to 300 s, and ultrasound amplitude, ranging from 50 to 100%, were evaluated.

After selection of the formulation with the lowest predicted volume-weighted mean droplet diameter, the effects of ascorbic acid and α-tocopherol on oxidative stability were evaluated. Ascorbic acid was added to the aqueous phase to obtain a final concentration of 20 mM. This concentration was selected as a literature-based benchmark following Uluata et al. [39]. α-Tocopherol was added directly to the n-3 PUFA concentrate at a concentration of 1.75 mg/g oil, following the oil-normalized concentration evaluated by Espinosa et al. [40]. These concentrations were used as single-level screening conditions and were not specifically optimized for the present emulsion formulation.

### 2.4. Experimental Design and Statistical Analysis

Response surface methodology (RSM) and central composite design (CCD) were used to study the effects of oil content (X1: 10–30% *w*/*w* of the total solids of the emulsion, which was 30% *w*/*w*), ultrasound emulsification time (X2: 60–300 s), and ultrasound amplitude (X3: 50–100%) on the volume-weighted mean droplet diameter, D[4,3], of the emulsions. D[4,3] was used as the response variable, Y. The design comprised 17 experimental conditions, including three replicates at the central point (Table 1). A second-order polynomial model was fitted to the experimental data to describe D[4,3] as a function of oil content, ultrasound emulsification time and ultrasound amplitude (Equation (1)):

Y = a_0_ + a_1_X_1_ + a_2_X_2_ + a_3_X_3_ + a_11_X_1_^2^ +a_22_X_2_^2^ + a_33_X_3_^2^ + a_12_X_1_X_2_ + a_13_X_1_X_3_ + a_23_X_2_X_3_ (1)

where Y is the volume-weighted mean droplet diameter, D[4,3]; a_0_ is the intercept; and a_i_, a_ii_ and a_ij_ are the linear, quadratic and interaction coefficients, respectively. The significance of the regression coefficients was evaluated from their F- and *p*-values. Analysis of variance was performed at a 95% confidence level to assess the significance and adequacy of the fitted model. D[4,3] was the only response variable included in the RSM model. The span value was used as a complementary descriptor of the width of the droplet-size distribution but was not included as a second optimization response. The experimental design and statistical analyses were performed using Statgraphics Centurion 19-x64 software (Statgraphics Technologies, Inc., The Plains, VA, USA).

### 2.5. Evaluation of the Emulsion Stability

Changes in the droplet-size distribution of the selected formulation were followed by laser diffraction using a Mastersizer 2000 instrument (Malvern Instruments Ltd., Malvern, UK). Measurements were conducted in triplicate at 25 °C immediately after preparation and after 2, 6, and 29 days of storage at ambient temperature in darkness. Droplet size was reported as the volume-weighted mean diameter, D[4,3]. Distribution width was additionally expressed as span, a dimensionless number defined by Equation (2):

(2) $$Span=\frac{D_{90}-D_{10}}{D_{50}}$$

where D_10_, D_50_, and D_90_ represent the droplet diameters below which 10%, 50%, and 90% of the cumulative dispersed-phase volume are found, respectively.

Physical destabilization was monitored without sample dilution by static multiple light scattering using a Turbiscan Lab Expert equipped with an automated ageing station (Formulaction Co., L’Union, France). An undiluted emulsion sample of 20 mL was placed in a cylindrical glass cell. Two synchronous optical sensors recorded the light transmitted through the sample at 180° relative to the incident beam and the light backscattered at 45°. Transmission and backscattering profiles were recorded as a function of cell height and storage time at 25 °C [41,42]. The emulsion was monitored for 24 days to detect gradual destabilization phenomena, including creaming and changes associated with droplet migration or growth. This extended observation period was not intended to represent the shelf life of the formulation.

### 2.6. Evaluation of Oxidative Stability of Emulsions

#### 2.6.1. Peroxide Value (PV)

Hydroperoxides were quantified by a ferric thiocyanate assay adapted from Shantha & Decker [43] and Uluata et al. [44]. A measured aliquot of emulsion was extracted with 1.5 mL of 2,2,4-trimethylpentane/2-propanol (3:1, *v*/*v*) by three consecutive 10 s vortexing cycles. Phase separation was achieved by centrifugation at 5000 rpm for 10 min (SORVALL ST16R, Thermo Fisher Scientific, Waltham, MA, USA). A 200 µL portion of the resulting organic phase was combined with 2.8 mL of methanol/butanol solution (2:1, *v*/*v*). Color development was initiated by adding 15 µL of 3.94 M ammonium thiocyanate and 15 µL of freshly prepared Fe^2+^ solution. The latter was obtained by mixing equal volumes of 0.132 M BaCl in 0.4 M HCl and 0.144 M FeSO_4_ and collecting the supernatant. After vortex mixing, the reaction mixtures were left for 20 min at room temperature without exposure to light. Absorbance was then recorded at 510 nm with a UV-VIS spectrophotometer (Hitachi U-2000, Tokyo, Japan). Hydroperoxide concentration was calculated from a calibration curve generated with cumene hydroperoxide and expressed relative to the oil content of the emulsion.

#### 2.6.2. TBARS Determination

Secondary oxidation products reactive toward thiobarbituric acid were measured using an assay adapted from Elias et al. [36] and Chityala et al. [37]. Depending on the expected concentration, 0.1–1.0 mL of emulsion was transferred to a glass tube and brought to a total volume of 1.0 mL with distilled water. Each diluted aliquot was mixed with 2.0 mL of TBA reagent containing 15% *w*/*v* trichloroacetic acid and 0.375% *w*/*v* 2-thiobarbituric acid in 0.25 M HCl. The sealed tubes were heated in boiling water for 15 min and subsequently allowed to cool at room temperature for 10 min. Precipitated material was removed by centrifugation at 5000 rpm for 15 min. Absorbance was recorded at 532 nm 10 min after centrifugation. TBARS concentration was obtained by comparison with a 1,1,3,3-tetraethoxypropane calibration curve and normalized to the amount of oil present in the sample.

## 3. Results and Discussion

### 3.1. n-3 PUFA Concentrate Fatty Acid Composition and Oxidation Values

The n-3 PUFA concentrate used in this study contained 73% DHA and 92% of n-3 PUFA, mostly as triglycerides (72 wt%), with minor quantities of diglycerides (11 wt%), monoglycerides (2 wt%), and ethyl esters (15 wt%).

The primary (peroxide value, PV) and secondary (*p*-anisidine value, AV) oxidation of this concentrate was analyzed, obtaining a PV of 3.79 ± 0.02 meq O_2_/kg and an AV of 11.9 ± 0.7, which are suitable for human consumption. In the TBARS assay, a value of 0.825 ± 0.033 mmol/kg was obtained for the TBARS content.

### 3.2. Influence of Oil Content, Ultrasound Emulsification Time and Amplitude on Emulsion Droplet Size—Optimal Conditions Using RSM

Response surface methodology was used to minimize the volume-weighted mean droplet diameter, D[4,3], of the n-3 PUFA concentrate emulsions by evaluating three formulation and processing variables: oil content, ultrasound emulsification time, and ultrasound amplitude. The experimental D[4,3] and span values are presented in Table 1. D[4,3] was fitted to the quadratic polynomial model described in Equation (1), whereas span was used as a complementary descriptor of the distribution width and was not included as an additional RSM response. The obtained model presented an R-squared of 96.2%, which indicates that the model fitted explains 96.2% of the variability in emulsion droplet size. All the coefficients of the quadratic model were statistically significant (*p* < 0.05), except for a_11_, a_33_, and a_13_ (Table 2). The F-value indicates that, for the range of values studied, the ultrasound emulsification time had a stronger influence on the emulsion droplet size than the oil content and amplitude (Table 2).

**Figure 1:**
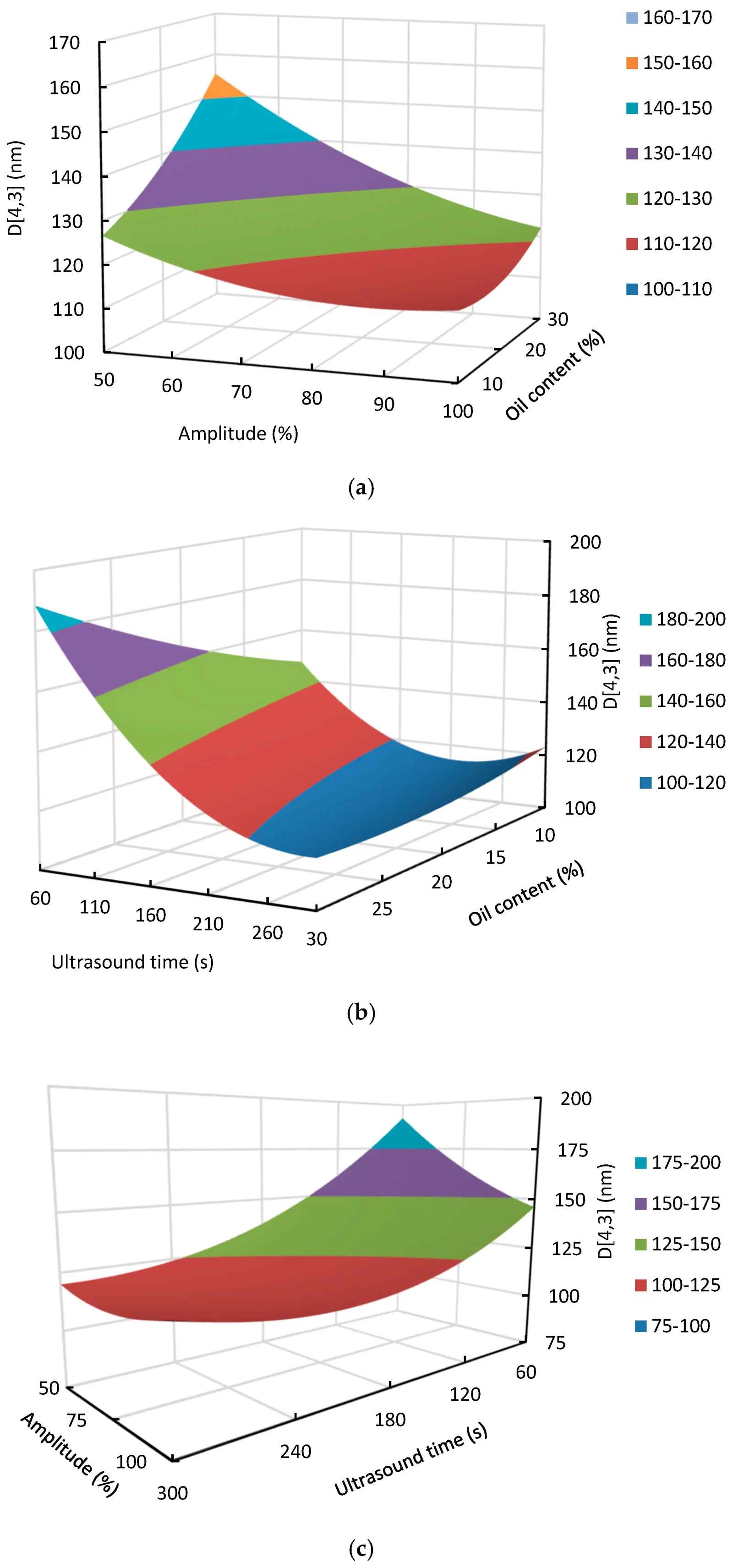
Response surface plot of the effects of the three variables (oil content, ultrasound emulsification time, and ultrasound amplitude) on the droplet size of the n-3 PUFA emulsions: (**a**) Oil content and amplitude at an ultrasound emulsification time of 180 s, (**b**) Oil content and ultrasound emulsification time at an ultrasound amplitude of 75%, (**c**) Ultrasound emulsification time and amplitude at an oil content of 20% *w*/*w*.

To study the effect of each variable on the emulsion droplet size, response surface plots of the quadratic polynomial model were generated by varying two of the three variables and keeping the third one at a fixed value, specifically at the central point of the range studied (Figure 1).

Figure 1a shows the effect of the oil content and ultrasound amplitude on the droplet size while maintaining a constant ultrasound emulsification time of 180 s. As shown in Figure 1a, the emulsion droplet size decreased when the ultrasound amplitude increased, which is typically observed in ultrasound emulsification processes [45]. Figure 1b shows the effect of the oil content and ultrasound emulsification time, with the amplitude of oscillation held constant at 75%. The droplet size of the emulsion decreased when the ultrasound emulsification time increased, which is in agreement with the literature [38,46]. The application of an excessive ultrasound emulsification time might result in the formation of an emulsion with a larger droplet size due to over-processing of the emulsion [47,48,49] which could lead to partial destabilization of the emulsion. These effects can also be observed in Figure 1c, which shows the effect of the ultrasound emulsification time and amplitude of oscillation while keeping the oil content constant at 20% *w*/*w*. Furthermore, under optimum conditions of ultrasound emulsification time and amplitude of oscillation (high values in both cases considering the studied range of each variable), small droplet sizes of emulsions were obtained throughout the selected range of oil content (10–30% *w*/*w*). This can be explained by considering that the effects of the time of ultrasound emulsification and amplitude of oscillation on the droplet size of the emulsion are stronger than the effect of the oil content (see Table 2).

According to the RSM model, the minimum D[4,3] was predicted to be 116 nm for an oil content of 20% *w*/*w* of the total solids, an ultrasound emulsification time of 180 s, and an ultrasound amplitude of 100%. Three emulsions were prepared under these conditions to validate the model prediction, resulting in an experimental D[4,3] of 121 ± 1 nm, with a corresponding span value of 1.16 ± 0.01. This formulation was selected for the subsequent storage experiments. Because a small initial droplet diameter alone does not demonstrate physical stability, changes in droplet-size distribution and destabilization during storage were subsequently evaluated by laser diffraction and static multiple light scattering, respectively.

### 3.3. Evaluation of the Physical Stability of the Selected Emulsion During Storage

The selected emulsion was stored at ambient temperature in darkness, and its droplet-size distribution was measured after 0, 2, 6, and 29 days. Day 29 was included as an extended endpoint approximately one month after preparation and should not be interpreted as a claimed shelf life. The absence of intermediate laser-diffraction measurements between days 6 and 29 limits the temporal resolution of the changes occurring during this interval.

**Figure 2:**
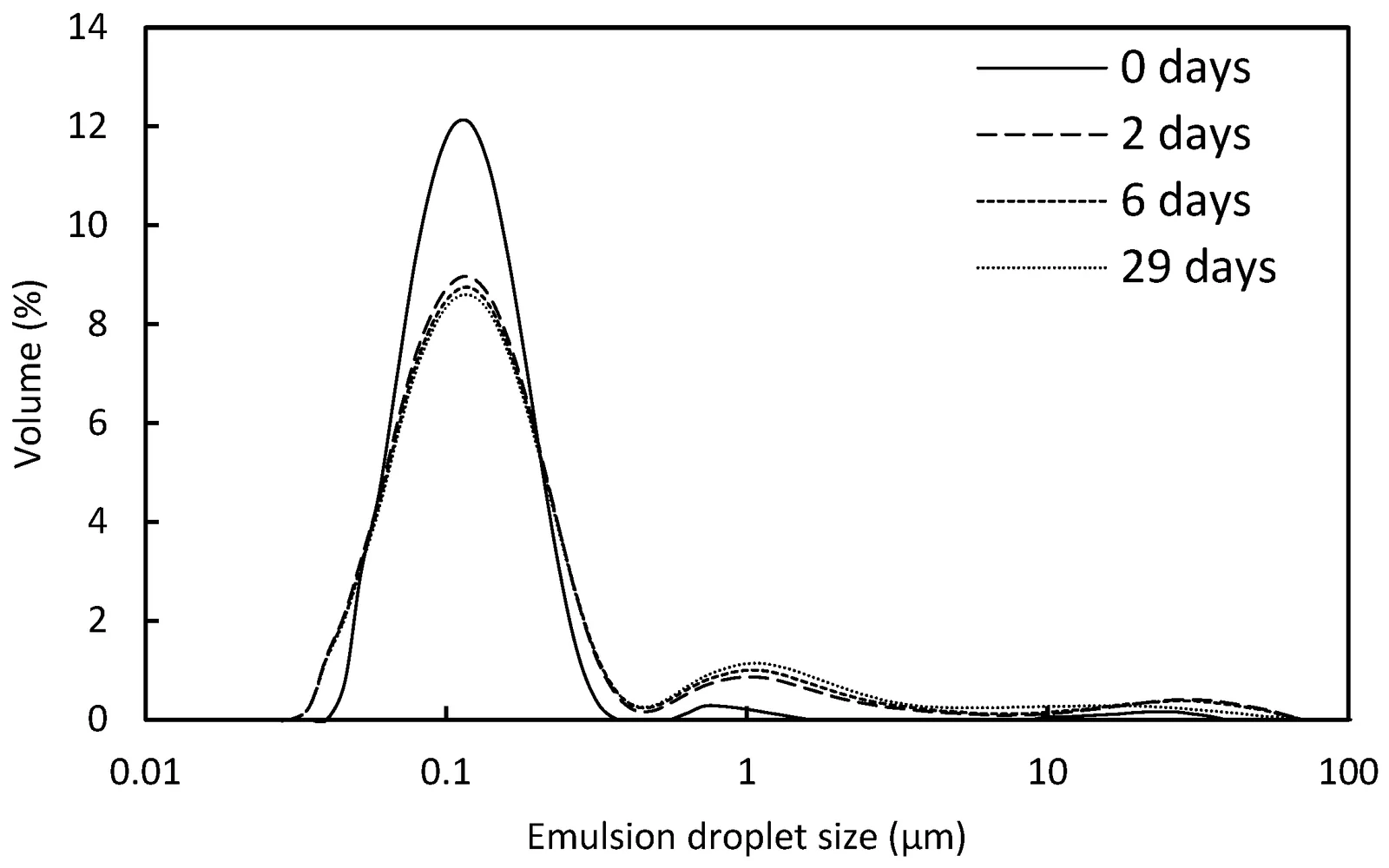
Droplet-size distribution of the selected emulsion prepared with an oil content equivalent to 20% *w*/*w* of the total solids, an ultrasound emulsification time of 180 s, and an ultrasound amplitude of 100%, after 0, 2, 6, and 29 days of storage at ambient temperature in darkness. Day 29 represents an extended storage endpoint and not an established shelf life.

Figure 2 shows the droplet-size distributions obtained at the different sampling times. The D[4,3] values at 0, 2, 6, and 29 days were 123, 136, 139, and 141 nm, respectively. The freshly prepared emulsion exhibited a predominantly monomodal distribution. After 2 d, a second population of larger droplets appeared, although its relative contribution was small compared with that of the main droplet population. This secondary population remained detectable at days 6 and 29. The moderate increase in D[4,3] indicates limited droplet growth during storage, but the appearance of the second population suggests that a small fraction of the droplets underwent aggregation or coalescence.

**Figure 3:**
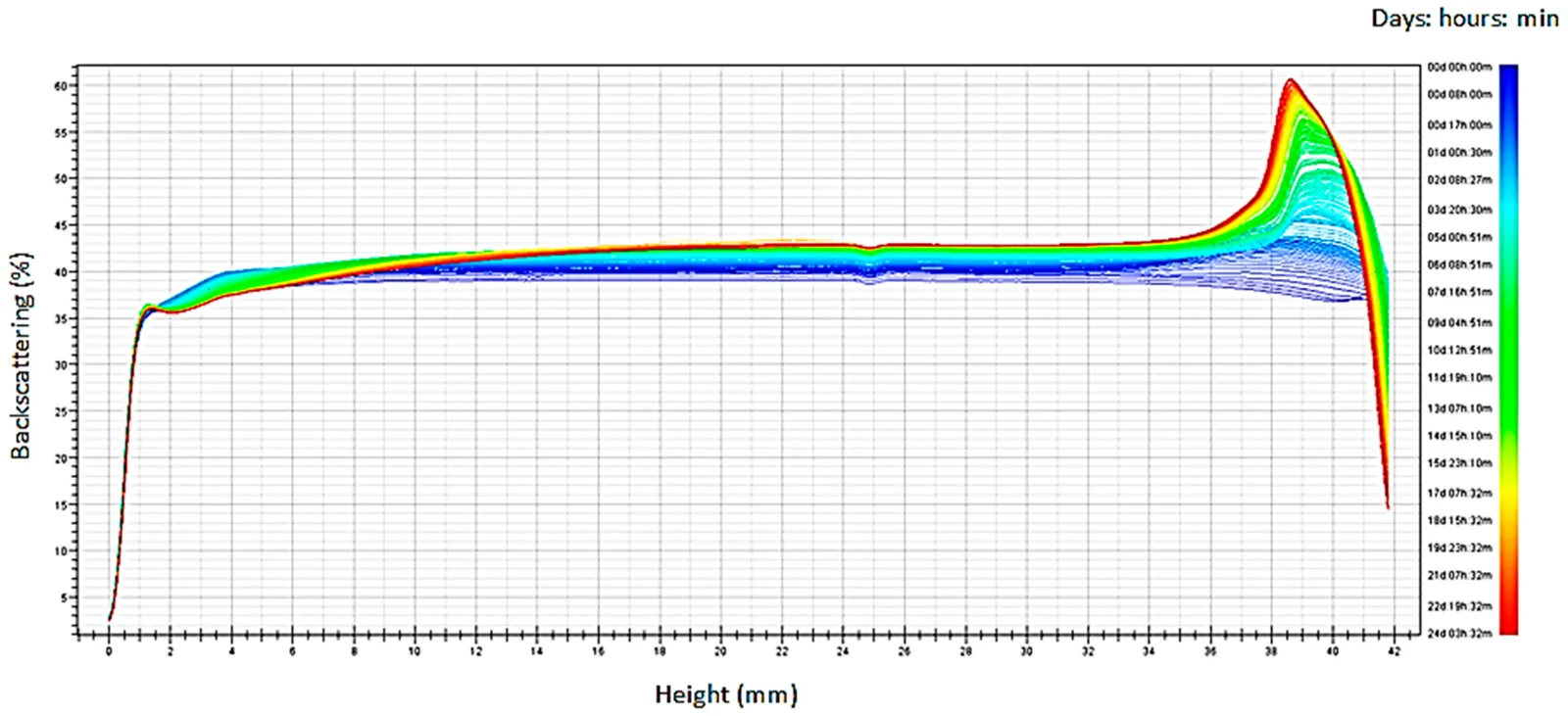
Backscattering profiles of the selected emulsion prepared with an oil content equivalent to 20% *w*/*w* of the total solids, an ultrasound emulsification time of 180 s and an ultrasound amplitude of 100%, during 24 days of storage at 25 °C. The monitoring period was selected to detect gradual physical destabilization and does not represent an established shelf life.

The physical stability of the selected emulsion was also evaluated by static multiple light scattering during 24 days of storage at 25 °C (Figure 3). An increase in backscattering in the upper region of the measurement cell became apparent from approximately day 6, indicating upward migration of the dispersed droplets and the onset of creaming. A slight increase in backscattering was also observed in the middle region of the cell, consistent with the moderate increase in droplet size detected by laser diffraction. Therefore, although the main droplet population remained relatively small during storage, the Turbiscan profiles demonstrate that the formulation was not completely resistant to gravitational destabilization.

### 3.4. Evaluation of the Oxidative Stability of the Selected Emulsion

The oxidative stability of the selected emulsion was evaluated by monitoring peroxide value (PV), as an indicator of primary lipid oxidation, and TBARS, as an indicator of secondary oxidation, during storage at ambient temperature in darkness.

In preliminary experiments, the n-3 PUFA concentrate was handled under ambient atmosphere during emulsion preparation. The initial PV and TBARS values of the resulting emulsion were 12.6 ± 1.0 meq O_2_/kg oil and 1.45 ± 0.07 mmol/kg oil, respectively. It should be noted that the initial peroxide value of the emulsion was higher than the recommended limit for human consumption, which is 5 meq O_2_/kg oil [50]. The PV remained approximately constant until day 5 and subsequently increased, whereas TBARS remained relatively constant until approximately day 6 before increasing. These preliminary results indicated that exposure of the highly unsaturated concentrate to atmospheric oxygen during handling markedly affected its initial oxidation state. Therefore, all subsequent opening and handling of the n-3 PUFA concentrate were performed under an N_2_ atmosphere.

**Figure 4:**
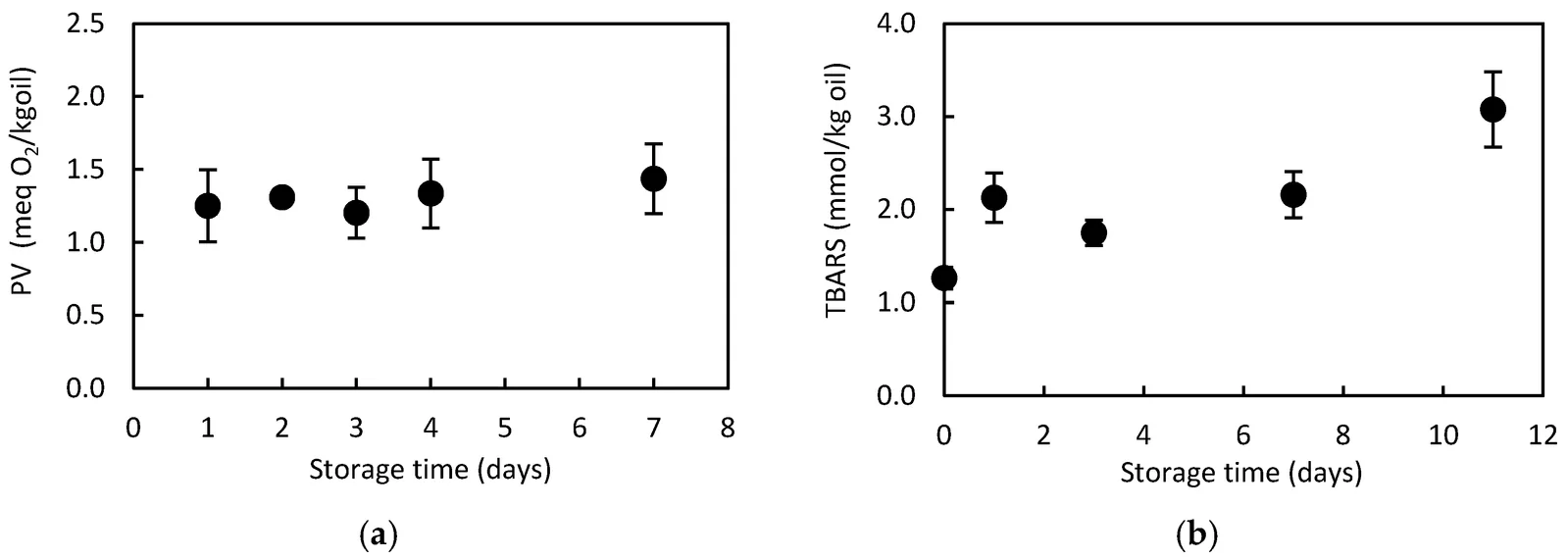
Oxidative stability of the selected n-3 PUFA concentrate emulsion stored at ambient temperature in darkness: (**a**) Peroxide value, determined at days 0, 1, 2, 3, 4, and 7; and (**b**) TBARS value, determined at days 0, 1, 3, 7, and 11.

Figure 4 shows the PV and TBARS values of the selected emulsion prepared using the n-3 PUFA concentrate handled under N_2_. PV was determined at days 0, 1, 2, 3, 4 and 7, whereas TBARS was determined at days 0, 1, 3, 7, and 11. The two oxidation indicators were therefore obtained according to separate sampling schedules, and direct point-by-point comparisons can only be made at the common sampling times. The PV remained low throughout the evaluated period, with values between approximately 1.1 and 1.4 meq O_2_/kg oil. In contrast, TBARS remained relatively constant through day 7 and increased at the final sampling point. The lower PV of the emulsion compared with that initially determined for the bulk n-3 PUFA concentrate should be interpreted cautiously because the samples were subjected to different handling conditions before analysis. Therefore, this difference cannot be attributed exclusively to the emulsification process. Overall, the formulation limited the accumulation of primary and secondary oxidation products during the initial storage period. Nevertheless, the increase in TBARS during extended storage indicates that the formulation delayed, rather than completely inhibited, lipid oxidation.

### 3.5. Effect of the Addition of Different Antioxidants to the Optimal Emulsion: Evaluation of Oxidative Stability

#### 3.5.1. Addition of Ascorbic Acid

Ascorbic acid (AA) was added to the aqueous phase of the optimal emulsion at a final concentration of 20 mM to decrease lipid oxidation rates because of the ability of AA to scavenge oxygen and inhibit lipid oxidation. This concentration was selected as a literature-based benchmark following Uluata et al. [39], who evaluated AA concentrations between 2.5 and 20 mM in oil-in-water emulsions containing 1% *w*/*w* soybean oil and reported greater inhibition of lipid oxidation at 20 mM than at the lower concentrations.

**Figure 5:**
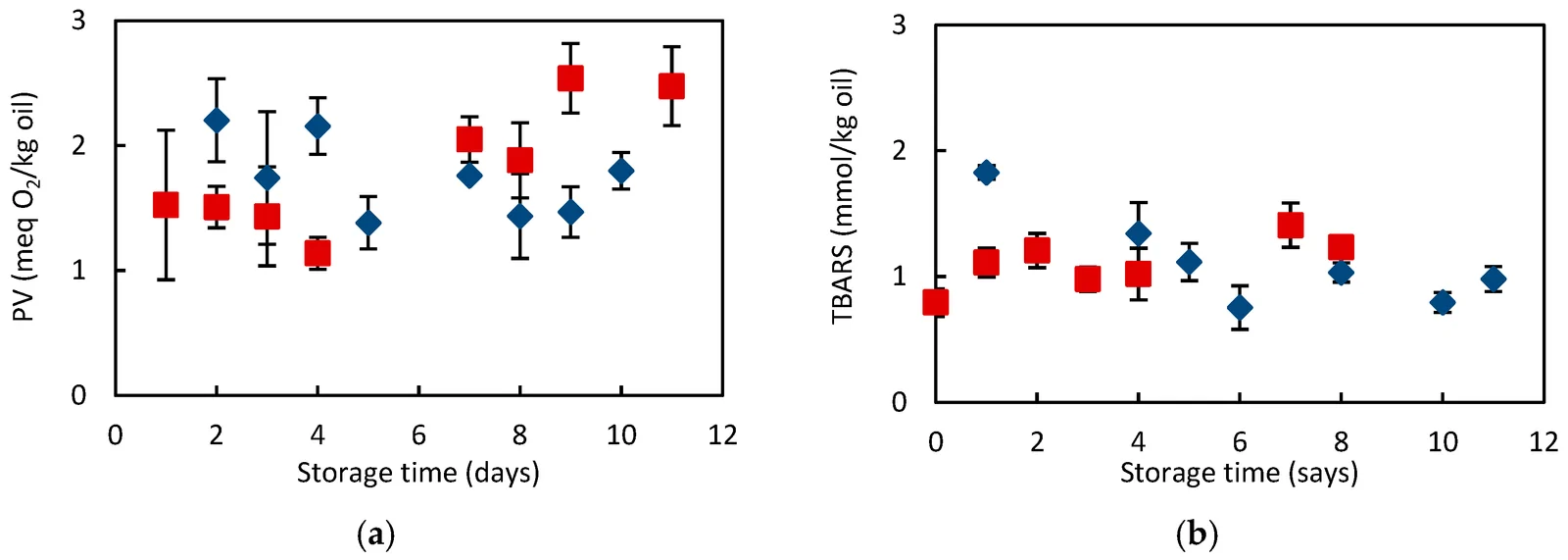
Oxidation stability of the selected n-3 PUFA concentrate emulsion with addition of AA over time, stored with N_2_ atmosphere and 4 °C (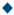) and stored at ambient temperature in darkness (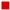), (**a**) Peroxide value (PV) and (**b**) TBARS value.

Figure 5 shows the PV and TBARS values of the AA-containing emulsion during storage. One aliquot was stored under N_2_ at 4 °C, whereas a second aliquot was stored at ambient temperature in darkness. Similar primary oxidation profiles were observed under both storage conditions, although slightly lower PV were obtained for the emulsion stored under N_2_ at 4 °C, consistent with the influence of temperature and oxygen availability on lipid oxidation [51,52]. TBARS values remained relatively low and constant throughout the evaluated storage period under both conditions. These values were slightly higher than the initial TBARS value of the n-3 PUFA concentrate, 0.825 ± 0.033 mmol/kg oil, but lower than those observed for the control emulsion during extended storage.

The primary and secondary oxidation values remained constant over time in both aliquots, indicating that this formulation of the highly rich n-3 PUFA concentrate used in this study as an oil-in-water emulsion with the addition of ascorbic acid was suitable for delaying or inhibiting lipid oxidation without the necessity of storing the optimal emulsion with N_2_ and at 4 °C, just storing the selected emulsion at ambient temperature and darkness. Uluata et al. [39] investigated the ability of AA to decrease oxygen concentrations and inhibit lipid oxidation in oil-in-water emulsions of commercial soybean oil using Tween 20 as an emulsifier. They obtained constant and low primary and secondary oxidation values over time, as presented in this study, confirming that lipid oxidation can be inhibited by the addition of AA.

#### 3.5.2. Comparative Effect of Ascorbic Acid and α-Tocopherol on Oxidative Stability

**Figure 6:**
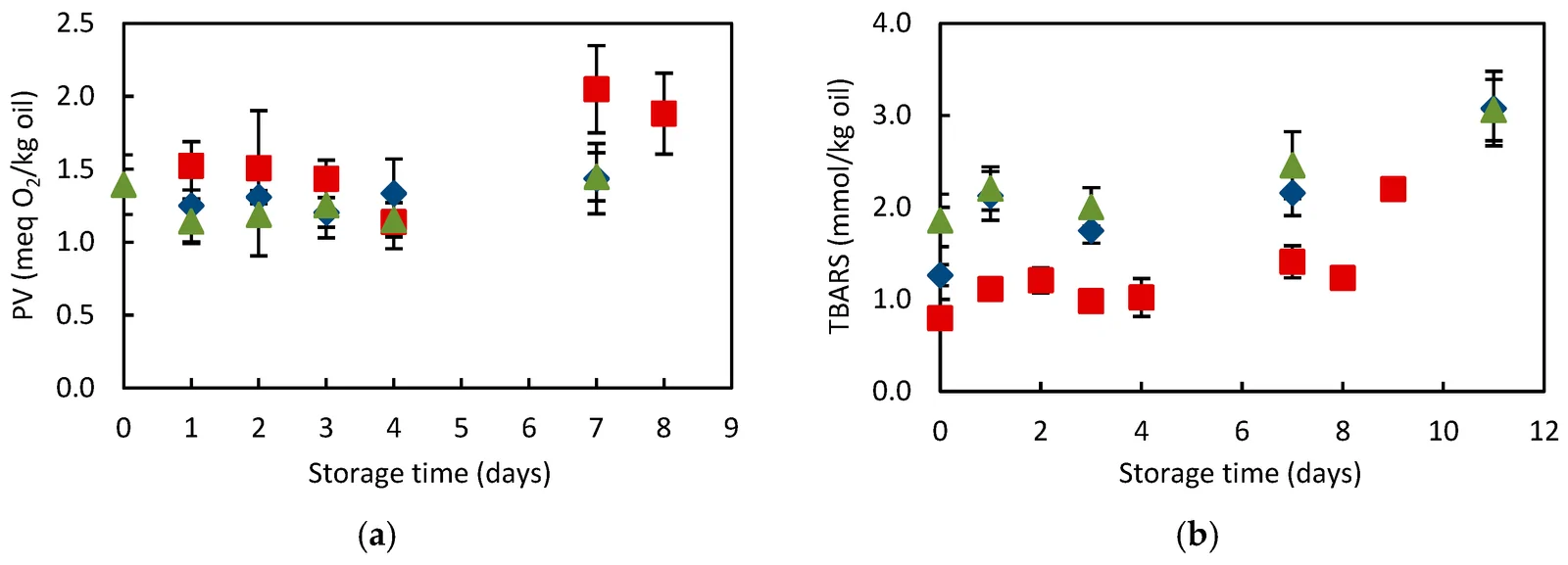
Comparative oxidative stability of the control emulsion (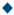), the emulsion containing 20 mM AA (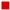) and the emulsion containing 1.75 mg α-tocopherol/g oil (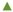) during storage at ambient temperature in darkness: (**a**) Peroxide Value (PV) and (**b**) TBARS value.

Figure 6 compares the oxidative stability of the selected emulsion prepared without added antioxidants, referred to as the control emulsion, and with the addition of either AA or α-tocopherol. AA was added to the aqueous phase at a final concentration of 20 mM, whereas α-tocopherol was incorporated into the oil phase at 1.75 mg/g n-3 PUFA concentrate. All emulsions were prepared under the same conditions and stored at ambient temperature in darkness. As shown in Figure 6a, peroxide values (PV) remained low and relatively constant throughout storage for the three formulations. No apparent differences were observed between the control emulsion and the emulsions containing AA or α-tocopherol. Therefore, under the conditions evaluated, antioxidant addition did not produce a measurable reduction in the accumulation of primary oxidation products.

Differences were observed in the formation of secondary oxidation products (Figure 6b). The control emulsion and the emulsion containing α-tocopherol exhibited similar TBARS profiles, with an increase during extended storage. Thus, the addition of α-tocopherol at 1.75 mg/g oil did not produce an apparent improvement in oxidative stability compared with the control. Espinosa et al. [40] reported a similar trend in their results for PV and TBARS values of a control emulsion of Echium oil using Tween 20 as a surfactant and the same emulsion with the addition of the same concentration of α-tocopherol as in this work (1.75 mg α-tocopherol/g oil).

The effectiveness of antioxidants in oil-in-water emulsions depends on several factors, including antioxidant concentration, the molecular environment, the composition and structure of the interfacial layer, and interactions with other components present in the system [51,52]. Consequently, the lack of effectiveness of α-tocopherol in the present formulation cannot be attributed solely to its lipophilic character or to its localization within the oil droplets. Other factors, such as its effective concentration in the interfacial region, the characteristics of the interface formed by OSA-modified starch, its possible degradation during processing or storage, and the presence of endogenous antioxidant or pro-oxidant compounds in the commercial n-3 PUFA concentrate, may also have influenced its activity. However, these factors were not specifically evaluated in the present study.

In contrast, the emulsion containing AA showed lower TBARS values than the control and α-tocopherol-containing emulsions during storage. The apparent protective effect of AA may be associated with its activity in the continuous aqueous phase, particularly its ability to consume dissolved oxygen and scavenge reactive species. Uluata et al. [39] demonstrated that AA concentrations ranging from 2.5 to 20 mM reduced dissolved oxygen and delayed the formation of secondary oxidation products in oil-in-water emulsions. This mechanism is consistent with the lower accumulation of TBARS observed in the AA-containing emulsion. However, dissolved oxygen, transition-metal content, and AA degradation were not measured; therefore, this explanation should be regarded as a plausible interpretation rather than a mechanism directly demonstrated by the present results. Overall, the addition of 20 mM AA delayed the accumulation of secondary lipid oxidation products under the conditions evaluated. Further concentration-response experiments would be required to establish the optimum AA concentration for this formulation.

## 4. Conclusions

The formulation of n-3 PUFA concentrate (a commercial fish-oil concentrate with 92% *w*/*w* total n-3 PUFA and 73% *w*/*w* DHA) using OSA-modified starch as carrier material and ultrasound-assisted emulsification was developed in this work. Response surface methodology identified an oil content equivalent to 20% *w*/*w* of the total solids, an ultrasound emulsification time of 180 s, and an ultrasound amplitude of 100% as the conditions that minimized the volume-weighted mean droplet diameter. The experimentally obtained D[4,3] under these conditions was 121 ± 1 nm. The mean droplet diameter increased only moderately during 29 days of storage. Nevertheless, static multiple light scattering detected creaming from approximately day 6, demonstrating that a small initial droplet diameter did not prevent gravitational destabilization. The present formulation should therefore not be described as completely physically stable throughout the evaluated storage period.

During storage at ambient temperature in darkness, PV remained low over the period evaluated, whereas TBARS increased during extended storage. The addition of α-tocopherol at 1.75 mg/g oil did not produce an apparent improvement in oxidative stability compared with the control emulsion. In contrast, the addition of 20 mM AA reduced TBARS accumulation, indicating that AA delayed the formation of secondary lipid oxidation products under the conditions studied. Because only one concentration of each antioxidant was evaluated, these results do not establish their optimum concentrations for the present formulation.

The developed emulsion represents a proof-of-concept formulation with potential application as an omega-3-enriched ingredient for aqueous or semi-aqueous food products. However, its commercial application requires further evaluation in specific food matrices, including sensory acceptability, long-term physical and oxidative stability, microbiological stability, and interactions with other food components. Process scale-up and optimization of antioxidant concentration should also be investigated. In addition, zeta potential, microscopic morphology, and the amount of non-emulsified oil should be determined to provide a more complete characterization of the interfacial and structural properties of the system.

## Figures and Tables

**Table 1:** Central composite design and experimental volume-weighted mean droplet diameter, D[4,3], and span values of the n-3 PUFA emulsions.

Emulsion	Oil Content (X_1_, % *w*/*w*)	Ultrasound Time (X_2_, s)	Amplitude (X_3_, %)	D[4,3] (Y, nm)	Span
1	20	300	75	118 ± 3	1.19 ± 0.01
2	20	180	100	122 ± 4	1.15 ± 0.01
3	10	300	50	122 ± 2	1.14 ± 0.01
4	10	180	75	120 ± 2	1.26 ± 0.02
5	10	60	100	133 ± 3	1.69 ± 0.02
6	30	300	50	129 ± 2	1.49 ± 0.01
7	30	60	100	165 ± 6	2.32 ± 0.02
8	20	180	75	124 ± 2	1.26 ± 0.01
9	20	180	75	126 ± 2	1.33 ± 0.01
10	10	60	50	172 ± 6	2.87 ± 0.11
11	10	300	100	130 ± 2	2.62 ± 0.04
12	20	180	75	127 ± 2	1.38 ± 0.01
13	20	60	75	157 ± 5	2.21 ± 0.04
14	30	60	50	229 ± 10	3.42 ± 0.12
15	30	180	75	124 ± 4	1.51 ± 0.01
16	30	300	100	117 ± 2	1.27 ± 0.03
17	20	180	50	125 ± 3	1.56 ± 0.01

**Table 2:** Analysis of variance (ANOVA) of the regression coefficients of the quadratic Equation (1) for the droplet size of n-3 PUFA concentrate emulsions.

Polynomial Coefficient	Polynomial Coefficient Value	F-Value	*p*-Value
a_0_	259.993		
a_1_	2.9726	10.49	0.0143
a_2_	−0.7844	79.8	0.0000
a_3_	−1.9122	16.76	0.0046
a_11_	0.0342	0.43	0.5316
a_22_	0.0013	13.28	0.0082
a_33_	0.0079	0.9	0.3752
a_12_	−0.0099	15.63	0.0055
a_13_	−0.0225	3.51	0.1033
a_23_	0.0041	16.97	0.0045

## Data Availability

The original contributions presented in the study are included in the article, further inquiries can be directed to the corresponding authors.
